# Malaysia’s Health Leadership in ASEAN: Driving Innovation, Reform and Future-Ready Health Systems

**DOI:** 10.21315/mjms-06-2026-s03

**Published:** 2026-08-31

**Authors:** Ismuni Bohari

**Affiliations:** 1 Chair, Senior Officials Meeting on Health Development (SOMHD), ASEAN; 2 Deputy Director General of Health (Public Health), Ministry of Health Malaysia, 62590 Putrajaya, Malaysia

**Keywords:** ASEAN, health leadership, health systems, regional cooperation, public health

## Abstract

Malaysia continues to champion stronger public health leadership in ASEAN by driving health transformation, resilience and regional solidarity. Through a future-ready approach focused on prevention, health security, innovation and sustainable health systems, Malaysia is shaping a healthier ASEAN with greater accessibility, equity and people-centred care, empowering communities, strengthening partnerships and advancing collective action to safeguard the wellbeing of all towards a truly Healthy ASEAN.

## Introduction

In today’s rapidly evolving and complex geopolitical environment, the era of “business as usual” is no longer sustainable. As Chair of the ASEAN Health Sector for 2025–2026, Malaysia has advocated for a strategic shift towards a more resilient, sustainable and future-ready ASEAN health system. Building on the Post-2015 Health Development Agenda 2021–2025 (1), ASEAN must realign priorities by focusing on high-impact initiatives that deliver meaningful outcomes, strengthen health security and maximise the value of collective resources. This transformation reflects ASEAN’s shared commitment towards achieving a Healthy ASEAN (1), a region where health systems are resilient, services are accessible and equitable, and communities are empowered to lead healthier lives. Through stronger collaboration, innovation and collective action, ASEAN will continue to build a sustainable health ecosystem capable of protecting its people and addressing future health challenges.

As Chair of the ASEAN Senior Officials Meeting on Health Development (SOMHD), Malaysia has taken a leading role in steering the transformation of the ASEAN Health Sector towards a more resilient, sustainable and future-ready health agenda. The Ministry of Health Malaysia convened the Collective Development Meeting and Workshop from 20–23 April 2026 in Kuala Lumpur, bringing together representatives from all ASEAN Member States to develop and align the ASEAN Health Sector Work Programmes 2026–2030 through an inclusive, collaborative and consensus-driven approach. The strategic direction was further strengthened during the Special SOMHD Meeting held from 6–8 May 2026 in Jakarta, Indonesia, chaired by Malaysia, where further refinement, reprioritisation and alignment of activities were undertaken, including the advancement of the ASEAN Strategic Health Agenda 2026–2030. This reflects ASEAN’s commitment to strengthening regional health cooperation through shared priorities, collective action and a renewed focus on initiatives that deliver meaningful impact to the people of ASEAN. Malaysia’s leadership underscores the importance of ASEAN centrality, solidarity and partnership in addressing evolving health challenges, including non-communicable diseases, climate-related health impacts, public health security threats and health inequities. Through continued collaboration among ASEAN Member States and partners, Malaysia remains committed to advancing a healthier, more equitable, resilient and future-ready ASEAN health ecosystem for the benefit of all communities across the region.

## From Foundations to Future: The Evolution of ASEAN Health Cooperation and Regional Health Solidarity

Since the establishment of the first ASEAN Health Ministers’ Meeting in 1980, ASEAN health cooperation has evolved into a strong regional platform built on political commitment, technical collaboration and shared responsibility (2). The Declaration of the ASEAN Health Ministers on Collaboration on Health, signed in Manila on 24 July 1980, laid the foundation for strengthening regional health collaboration, promoting self-reliance and integrating health as an essential component of socioeconomic development (2). Over the years, ASEAN health cooperation has continued to evolve, with the establishment of four ASEAN Health Clusters in 2016 to provide a structured framework in addressing regional health priorities (3):

- ASEAN Health Cluster 1: Promoting Healthy Lifestyle Focusing on prevention, health promotion and addressing lifestyle-related health challenges to support healthier communities across ASEAN.
- ASEAN Health Cluster 2: Responding to All Hazards and Emerging Threats Strengthening regional preparedness, surveillance, response capacity and coordination in managing public health emergencies and emerging health threats.
- ASEAN Health Cluster 3: Strengthening Health Systems and Access to Care Advancing resilient health systems, improving healthcare accessibility, promoting equity and ensuring sustainable delivery of quality health services.
- ASEAN Health Cluster 4: Ensuring Food Safety Enhancing regional cooperation, risk assessment and science-based approaches to ensure safe and secure food systems across ASEAN.

In addition to that, several subsidiary bodies exist within each of these ASEAN Health Clusters to support implementation of activities (3), such as the ASEAN Centre for Active Ageing and Innovation (ACAI) under AHC 1, the ASEAN Biological Threats Surveillance Centre ABVC, ASEAN Institute for Disaster Management (AIDHM) and ASEAN Centre for Public Health Emergencies and Emerging Diseases (ACPHEED) under AHC 2 (4). Malaysia’s leadership in the ASEAN EOC Network for Public Health Emergencies, spearheaded by the Ministry of Health’s Crisis Preparedness and Response Centre (CPRC), has established a robust regional infrastructure for disease control, technical capacity building, and cross-border crisis collaboration **(5)**. The ASEAN Risk Assessment Centre for Food Safety (ARAC) under AHC 4 is also another great example of Malaysia’s leadership. Hosted by the Ministry of Health Malaysia since 2014, the ARAC has successfully established a unified, science-based food safety framework across the region (6).

## Redefining Regional Cooperation: Advancing Collective Action Towards a Resilient and Future-Ready ASEAN Health Ecosystem

Health threats are increasingly recognised as transboundary challenges that require collective regional action (7). Within the ASEAN context, interconnected economies, dense populations, climate vulnerabilities and extensive cross-border mobility have heightened the potential for health risks to transcend national boundaries and rapidly evolve into regional concerns (7, 8). Addressing these complex challenges requires more than individual national responses; it demands sustained multilateral cooperation, coordinated preparedness and strengthened regional solidarity to safeguard ASEAN’s health security, stability and resilience (7).

Recognising the evolving complexity of global health challenges, Malaysia, under its 2025 ASEAN Chairmanship themed “Inclusivity and Sustainability”, has championed a strategic transformation of the ASEAN health agenda—moving beyond reactive pandemic response towards building resilient, sustainable, data-driven and equitable health systems. This vision emphasises stronger regional cooperation, enhanced preparedness, strengthened health system capacities and sustained partnerships with ASEAN Member States, dialogue partners and development partners to ensure the continuity of essential health services and better health outcomes for all communities (9).

As part of Malaysia’s commitment to regional health security and resilience, the ASEAN Leaders’ Commitment on ASEAN Drug Security and Self-Reliance (ADSSR) was spearheaded in 2025 to strengthen pharmaceutical security across the region. Through this strategic framework, Malaysia continues to advocate for sustainable access to safe, affordable and quality-assured medicines, while addressing vulnerabilities in global supply chains and mitigating risks related to shortages of essential and life-saving medicines. Malaysia remains committed to advancing a resilient, self-reliant and equitable ASEAN health ecosystem, where innovation, partnership and collective action serve as the foundation towards achieving a Healthy ASEAN, a region where every community is empowered, health systems are prepared, and the wellbeing of all people is protected.

## Addressing the Rising Burden of Non-Communicable Diseases: Strengthening Prevention, Innovation and Health System Resilience

Traditionally, regional health security in ASEAN has focused on infectious disease control and epidemiological preparedness, particularly following major health crises such as severe acute respiratory syndrome (SARS) and COVID-19 (10). However, the region is now facing a dual health burden, with non-communicable diseases (NCDs), rising childhood obesity and population ageing emerging as critical challenges that require coordinated, multisectoral action (11, 12). Malaysia continues to advocate for stronger ASEAN collaboration in addressing NCD prevention through whole-of-government and whole-of-society approaches (11), while recognising the ageing population as not only a social responsibility but also an opportunity to strengthen the Silver Economy and future regional resilience (12).[Fig f1-01mjms3304_ed]

Malaysia has also taken a leading role in advancing lung health across ASEAN by promoting integrated respiratory care, tobacco control and early detection strategies (13). The successful tabling of the landmark resolution on integrated lung health, adopted at the 79th World Health Assembly in 2025 (13), established a global and regional framework to unify care pathways for tuberculosis (TB), chronic obstructive pulmonary disease (COPD), asthma and lung cancer. Through innovation such as Artificial Intelligence (AI)-supported chest X-ray screening and portable imaging technologies (14), Malaysia continues to strengthen equitable access to early diagnosis and care, reinforcing the commitment towards Universal Health Coverage and ensuring that no one is left behind (15).[Fig f2-01mjms3304_ed]

## Strategic Partnerships: Catalysing Collective Action Towards a Resilient and Sustainable Future

In the contemporary global health landscape, regional leadership is increasingly defined not merely by healthcare infrastructure, technological capability or manufacturing capacity, but by the ability to convene diverse stakeholders, facilitate strategic dialogue and foster meaningful cross-border collaboration (16). Within this context, Malaysia is strategically positioned to provide leadership in advancing ASEAN health cooperation by promoting collective action and strengthening partnerships among ASEAN Member States, dialogue partners and development partners (16). The complexity of emerging health challenges requires a collaborative and integrated approach that transcends traditional boundaries. Health initiatives within ASEAN can no longer be addressed through isolated efforts within individual Health Clusters alone; instead, they require cross-cutting collaboration through inter-pillar, inter-sectoral and inter-cluster mechanisms (17). Such an approach enables ASEAN to maximise resources, enhance policy coherence and deliver greater impact for the region. Moving beyond traditional donor-driven models, ASEAN has increasingly embraced a proactive and partnership-oriented approach in shaping its health agenda. Through strategic collaboration, ASEAN and its partners are working towards creating mutual value by aligning investments and technical support with shared regional priorities. This collective approach reinforces ASEAN’s ownership, strengthens health resilience and advances the vision of a sustainable, equitable and Healthy ASEAN.

## Conclusion

Malaysia’s tenure at the helm of ASEAN represents a significant milestone in advancing regional health diplomacy and shaping the future direction of ASEAN health cooperation. Through a strategic shift from short-term crisis response towards long-term sustainability, Malaysia has championed a broader and more inclusive approach to health security—encompassing infectious disease preparedness, NCDs, climate and environmental health risks, health inequities and emerging global challenges.

As Chair of the ASEAN Health Sector, Malaysia has played a pivotal role in strengthening regional collaboration through the development of a future-ready health agenda, promoting resilient, sustainable, data-driven and equitable health systems. By leveraging innovation, evidence-based policymaking and strategic partnerships, Malaysia continues to advocate for stronger health security, improved access to quality healthcare and responsive health systems that address the evolving needs of ASEAN communities. Malaysia has also advanced the principles of the One Health approach, recognising the interconnectedness between human, animal and environmental health. Through greater multi-sectoral collaboration, ASEAN is better positioned to address complex challenges including emerging infectious diseases, zoonotic threats, antimicrobial resistance, food safety and climate-related health impacts. This integrated approach reinforces the region’s preparedness and strengthens collective health resilience.

The journey towards a healthier ASEAN requires shared responsibility, regional solidarity and strong partnerships among ASEAN Member States, dialogue partners and development partners. Through collective action, innovation and cooperation, ASEAN can continue to build a future-ready health ecosystem that protects its people, strengthens resilience and ensures that economic progress is matched by improvements in health, wellbeing and quality of life. Ultimately, Malaysia remains committed to advancing the vision of a Healthy ASEAN, a region that is resilient, equitable, sustainable and prepared for the health challenges of tomorrow.[Fig f3-01mjms3304_ed]

## Figures and Tables

**Figure 1 f1-01mjms3304_ed:**
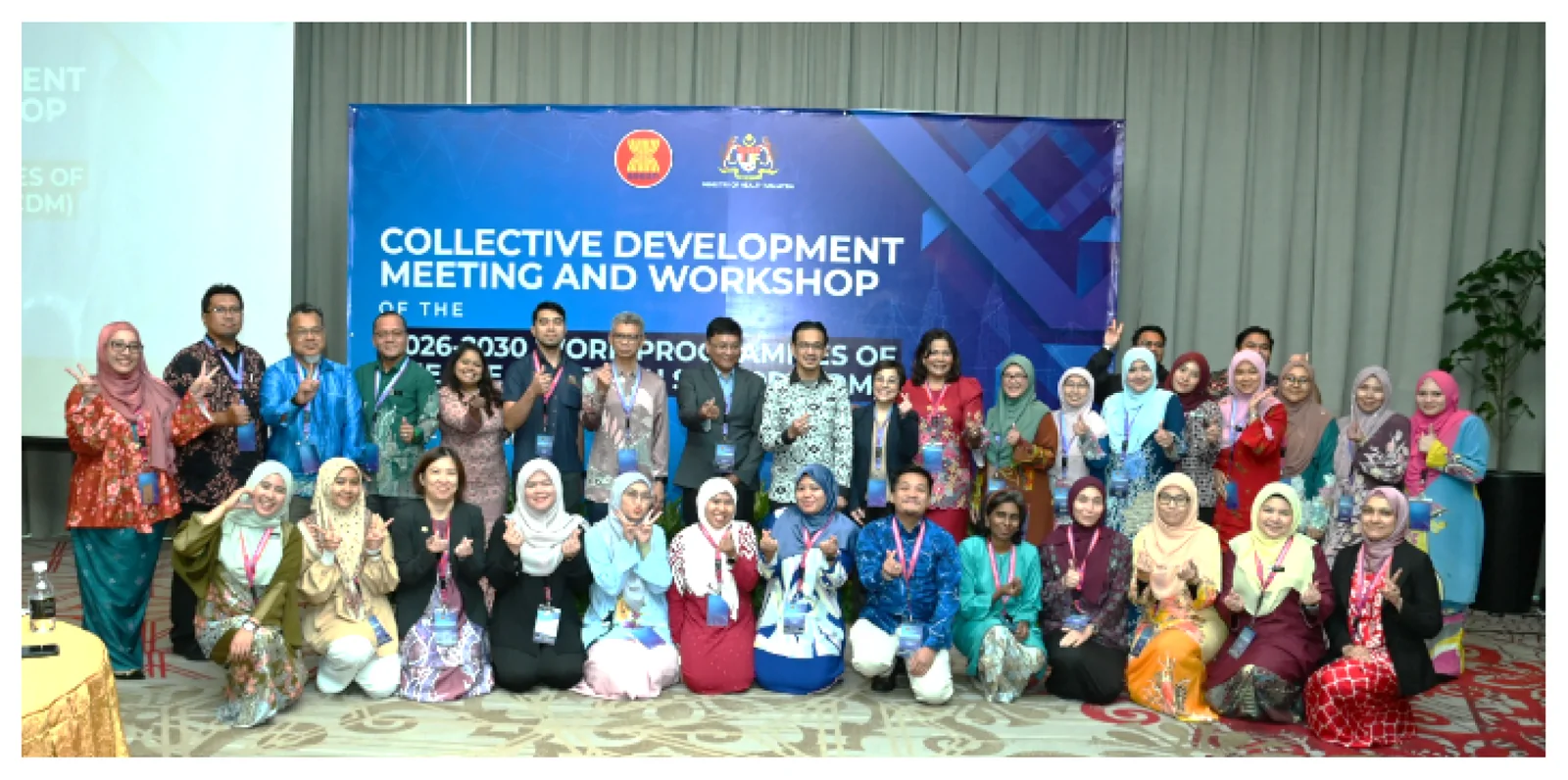
ASEAN Collective Development Meeting and Workshop held in Kuala Lumpur, Malaysia (20–23 April 2026), bringing together representatives from ASEAN Member States to develop the ASEAN Health Sector Work Programmes 2026–2030.

**Figure 2 f2-01mjms3304_ed:**
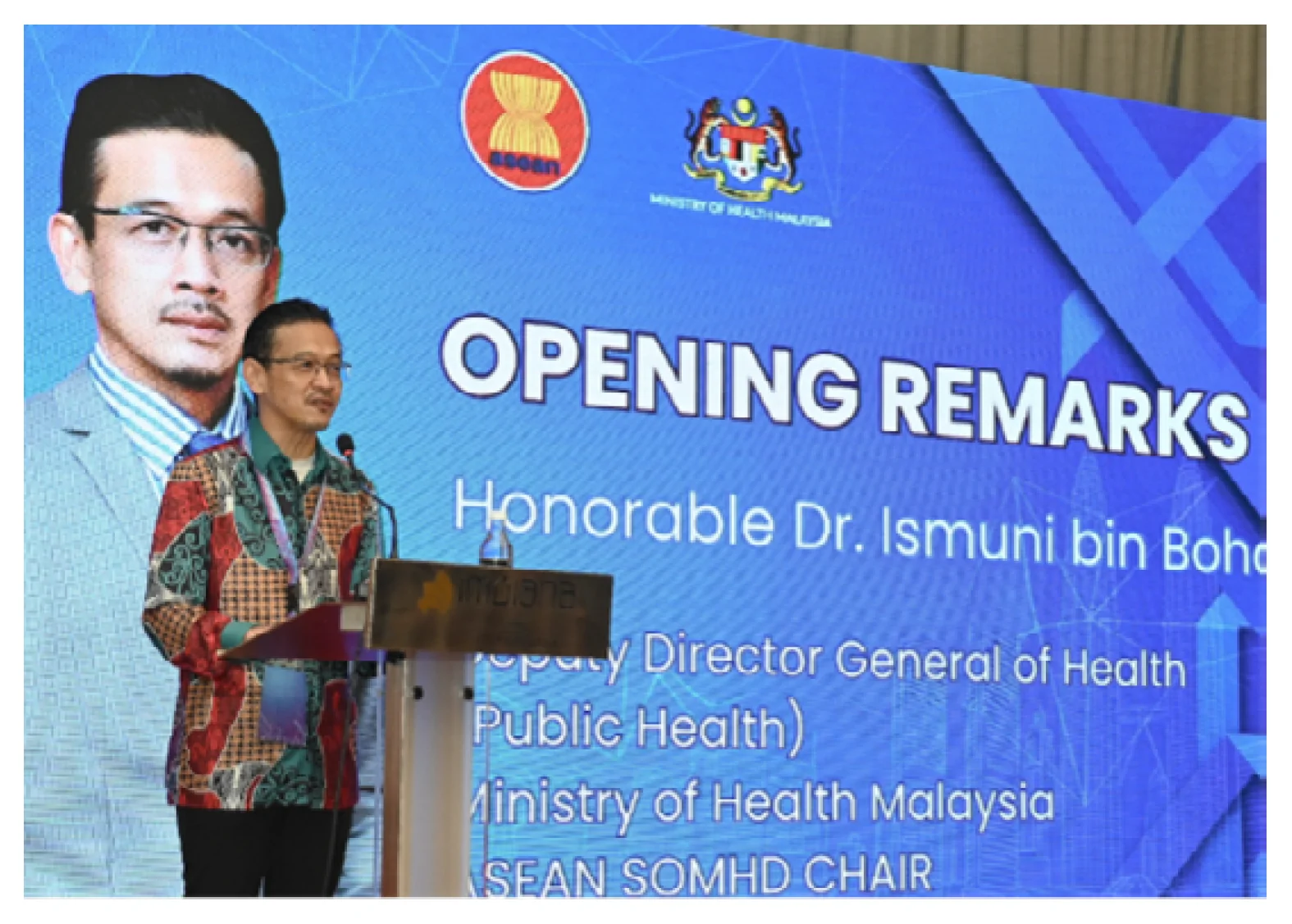
Opening address by the Chair of the ASEAN SOMHD, highlighting Malaysia’s commitment towards strengthening regional health cooperation.

**Figure 3 f3-01mjms3304_ed:**
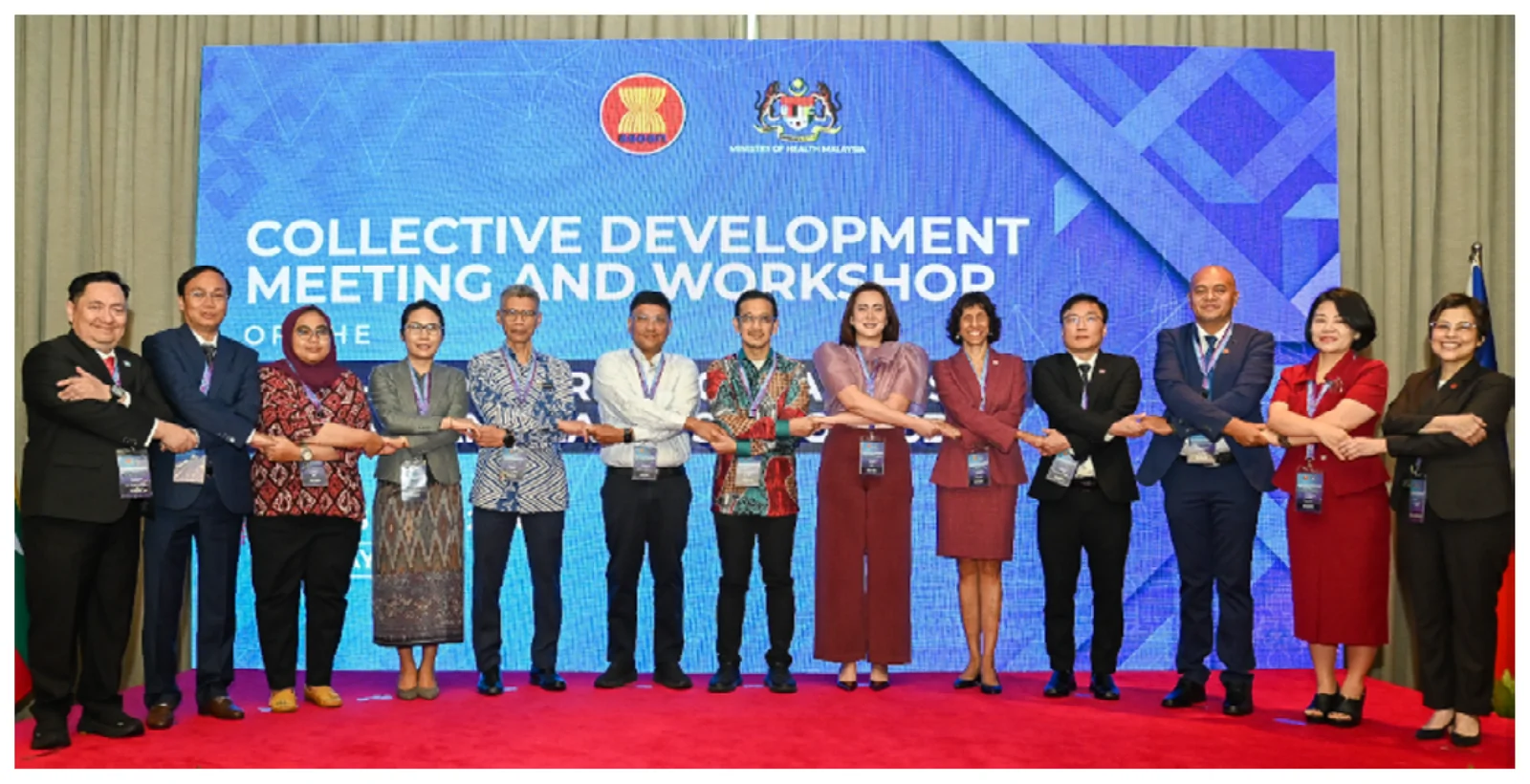
ASEAN delegates participating in the Collective Development Meeting and Workshop in Kuala Lumpur, reaffirming regional collaboration towards a resilient and future-ready ASEAN health ecosystem.
